# Extraction and Analysis of Impervious Surfaces Based on a Spectral Un-Mixing Method Using Pearl River Delta of China Landsat TM/ETM+ Imagery from 1998 to 2008

**DOI:** 10.3390/s120201846

**Published:** 2012-02-09

**Authors:** Yingbin Deng, Fenglei Fan, Renrong Chen

**Affiliations:** School of Geography, South China Normal University, Guangzhou, Guangdong 510631, China; E-Mails: bin19870521@gmail.com (Y.D.); chenrr87@yahoo.cn (R.C.)

**Keywords:** impervious surface area, linear spectral un-mixing, Pearl River Delta (PRD), percentage, change direction

## Abstract

Impervious surface area (ISA) is considered as an indicator of environment change and is regarded as an important input parameter for hydrological cycle simulation, water management and area pollution assessment. The Pearl River Delta (PRD), the 3rd most important economic district of China, is chosen in this paper to extract the ISA information based on Landsat images of 1998, 2003 and 2008 by using a linear spectral un-mixing method and to monitor impervious surface change by analyzing the multi-temporal Landsat-derived fractional impervious surface. Results of this study were as follows: (1) the area of ISA in the PRD increased 79.09% from 1998 to 2003 and 26.88% from 2003 to 2008 separately; (2) the spatial distribution of ISA was described according to the 1998/2003 percentage respectively. Most of middle and high percentage ISA was located in northwestern and southeastern of the whole delta, and middle percentage ISA was mainly located in the city interior, high percentage ISA was mainly located in the suburban around the city accordingly; (3) the expanding direction and trend of high percentage ISA was discussed in order to understand the change of urban in this delta; High percentage ISA moved from inner city to edge of urban area during 1998–2003 and moved to the suburban area that far from the urban area mixed with jumpily and gradually during 2003–2008. According to the discussion of high percentage ISA spatial expanded direction, it could be found out that high percentage ISA moved outward from the centre line of Pearl River of the whole delta while a high ISA percentage in both shores of the Pearl River Estuary moved toward the Pearl River; (4) combining the change of ISA with social conditions, the driving relationship was analyzed in detail. It was evident that ISA percentage change had a deep relationship with the economic development of this region in the past ten years. Contemporaneous major sport events (16th Asia Games of Guangzhou, 26th Summer Universidad of Shenzhen) and the government policies also promoted the development of the ISA. Meanwhile, topographical features like the National Nature Reserve of China restricted and affected the expansion of the ISA. Above all, this paper attempted to extract ISA in a major region of the PRD; the temporal and spatial analyses to PRD ISA demonstrated the drastic changes in developed areas of China. These results were important and valuable for land use management, ecological protection and policy establishment.

## 1. Introduction

Impervious surface is defined as the land surface which water cannot infiltrate. It is an anthropogenic feature, including roads, driveways, highways, sidewalks, outdoor basketball courts, rooftops, and so on. Impervious surface is considered as an indicator of environmental change and an important input parameter for the hydrological cycle simulation, water management and area pollution assessment [1]. Also, impervious surfaces are directly related to the water quality of surrounding lakes and streams for they affect the runoff of streams. Watersheds with large amounts of impervious surface may experience an overall decrease in groundwater recharge and base flow and an increase in storm flow and flood frequency [2]. Besides, it is concerned with energy balances and urban heat island effects [3].

Impervious surfaces can be extracted from various remote sensing images, high spatial resolution images such as QuickBird and IKONOS [4–6], medium spatial resolution images such as Landsat TM and Terra ASTER [7–12], and coarse resolution spatial resolution images such as DMSP-OLS and InSAR data [13–15].

The precision when assessing urban impervious surfaces using poor spatial resolution images is unsatisfactory because of the complexity of urban surface materials [8]. High spatial resolution images could interpret the surface materials clearly, but the shadows of tall building and clouds could obviously affect the precision of impervious surface mapping [8]. Besides, the cost of high spatial resolution images acquisition for large geographic areas was prohibitively expensive [3]. Therefore, different types of remote sensing images were selected for different applications. Medium spatial resolution images such as Landsat TM and ETM+ might be a better choice for the urban impervious surface mapping, because medium spatial resolution images are easy to obtain and the image’s precision is acceptable to be used for impervious surface mapping.

Lots of methods for extracting impervious surface were invented in recent years. Popularly, these include the decision tree method, supervised and un-supervised method, maximum likelihood method, neural network [4,6,16–18], regression tree model [19,20], linear spectral un-mixing method [21–23], non-linear spectral un-mixing method and fuzzy classifiers [24], Gaussian mixture discriminate analysis and so on.

Ridd assumed that the urban environment was covered by a linear combination of three components: vegetation, impervious surfaces and soil (V-I-S) [25]. In most cases, the three components of V-I-S were mixed together in a pixel of medium spatial resolution images. Consequently, per-pixel based methods were limited to extract the impervious surfaces accurately because of the dynamical changes in the urban environment and the complexity of the materials in a pixel. However, sub-pixel classification was more suitable for mixed pixel classification for it enabled us to quantify the percentage of each end-member of a pixel [7–9,11,26].

The linear spectral mixture (LSM) model was widely used for un-mixing mixed pixels. The spectral signatures in one pixel were assumed to be a linear combination with their proportions as weighting factors in the LSM model. The LSM model was an excellent approximation technique which was suitable for dealing with the spectral mixture problem, because it produced a set of maps that represented the abundance of each component. The LSM model included the fully constrained LSM method and the unconstrained LSM method. The fully constrained LSM method required that the signature abundance must met two requirements: (1) sum-to-one constraint; (2) non-negativity constraint. Compared with the fully constrained LSM method, the unconstrained LSM did not need to meet both of the above requirements. However, the unconstrained LSM methods might not represent the accurate abundance fractions [27]. A fully constrained LSM method was used for un-mixing mixed-pixels in this study. In the LSM model, the selection of end-members was very important and was a key step to extracting the impervious surfaces. Mountrackis *et al.* [28] assessed the incorporation of road structural information in the classification process of impervious surface areas. Franke *et al.* [29] gave a MESMA method to map four levels of complexity ranging from the simplest level consisting of only two classes.

Spatial-temporal analysis is an effective method to understand the change of geographical phenomenon. It has been applied to many research fields such as land use and land cover change [23,30–32], medical geography [33,34], hydrology [35,36], forest environment detecting [37], *etc*. Its results also depict the development trends and rates of change from the point of view of different years, therefore, spatial-temporal analysis could be a useful method to detect the change of impervious surfaces.

Previous researchers have done a lot of studies on the impervious surfaces of small scale regions, however, few of them paid attention to large scale impervious surfaces or suburban areas by using Landsat images. Furthermore, most of the researchers who have discussed the impervious surface of the Pearl River Delta (PRD) were paying attention to the small scale areas rather than the whole delta. Fan discussed the Land use and Land Change (LULC) of the core PRD using Landsat images, but in his research, traditional methods were applied and the data was obtained only based on pixels. Up to now, the changed area, expended direction and slope of impervious surface percentage of the core PRD have not been discussed based on sub-pixel methode. This study extracted the impervious surface of the PRD using a linear spectral un-mixing method. Besides the area of ISA, ISA distribution and high percentage ISA’s distribution of the core PRD are discussed.

The objectives of this paper included: (1) extracting the impervious surface of 1998, 2003 and 2008 respectively; (2) counting the area and analyzing the rate of change of the impervious surface of the core PRD from 1998 to 2008; (3) describing the distribution of the ISA; (4) analyzing the change direction of high percentage ISA of the core PRD from 1998 to 2008; (5) discussing the reasons that influenced the distribution of ISA.

## 2. Study Area and Dataset

**Figure 1.**
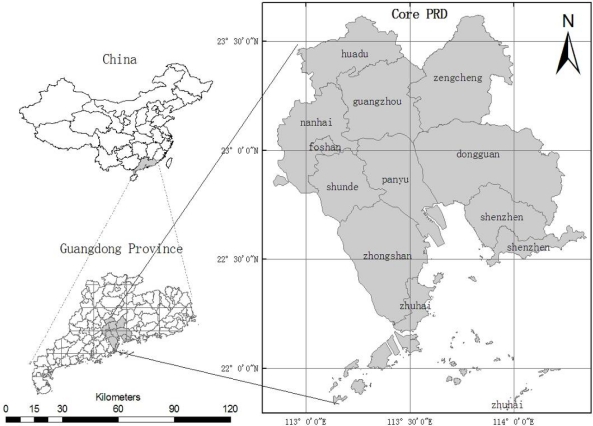
The location of study area.

The study area of this paper (Figure 1) was the core region of the PRD, which is located between latitude 22°N and 23.6°N, and between longitudes 112.6°E and 114.4°E, and includes 12 cities/counties such as Shenzhen, Baoan, Dongguan, Guangzhou, Huadu, Zengcheng, Panyu, Chancheng, Nanhai, Shunde, Zhongshan and Zhuhai. The area covers 21,388.1 km^2^ with a population of 19,844,000 [31]. It has a subtropical climate with an average annual temperature between 21 and 23 °C, and the average precipitation from 1,600 to 2,600 mm. About 80% of the rainfall comes in April to September with the impact of the East Asian Monsoonal circulation. It is obvious that the climate of the PRD includes a dry season and a wet season. The dry season is from April to September, the wet season is from October to February of the following next year.

The GDP of the PRD has exhibited a double-digit growth rate over the past two decades; it has appeared as one of the most advanced regions in the nation [38,39]. The GDP of the study area was 78.31% of the whole PRD in 1998, 81.4% in 2003 [31]. The grow rate of the GDP was related to ongoing the urbanization and the ISA was one of the indicators of this urbanization, so it was necessary to detect the ISA of the core PRD to understand the urbanization of the study area.

Two scenes of Landsat-5 Thematic Mapper (TM) Images (122/44, 122/45) acquired on 22 December 1998, two scenes of TM images (122/44, 122/45) acquired on 10 December 2008 and two scenes of Landsat-7 Enhanced Thematic Mapper Plus (ETM+) images (122/44, 122/45) acquired on 10 January 2003 were used in this research. All the Landsat images were rectified to the Transverse Mercator projection system. A systematical geometric correction and radiometric correction were performed to the images using the calibration parameter file (CPF) released by the United States Geological Survey Earth Resources Observation Systems Data Center, before the delivery. TM images of 1998 and 2008 were further registered and rectified to the mosaic ETM+ image of 2003 by the “image to image” method including the RST method (rotation, scaling, and translation, one of the warping methods) and the nearest neighbor image re-sampling algorithm.

## 3. Methods

### 3.1. Image Masking

Image masking is a useful way to separate water information from TM images. The spectral characteristics of water are similar to those of a low albedo impervious object. Water was hard to separate from the low albedo end-member which would affect the end member un-mixing results, so it was necessary to mask the water area before MNF transformation. In this research, the normalized difference water index (NDWI) [40] was applied to mask the water information, NDWI is a satellite-derived index from the Near-Infrared (NIR) and Short Wave Infrared (SWIR) channels, and it was a good indicator for vegetation liquid water and was less sensitive to atmospheric scattering effects than NDVI (Normalized Difference Vegetation Index). NDWI was expressed with the following formula:

(1) $$\mathrm{NDWI}=\frac{\left(p_{\mathrm{NIR}}-p_{\mathrm{SWIR}}\right)}{\left(p_{\mathrm{NIR}}+p_{\mathrm{SWIR}}\right)}$$

where NDWI means Normalized Difference Water Index; p_NIR_ and p_SWIR_ were the reflectance of NIR band and SWIR band, respectively.

In this paper, water masking from the TM images involves three steps: (1) calculate NDWI; (2) set a threshold of NDWI to extract water area; (3) mask the water area of all images.

### 3.2. Linear Spectral Mixture Analysis (LSMA)

The urban landscape is a complex assemblage was composed of anthropogenic feature and natural features, such as grass, trees, water and different kinds of impervious surfaces [21]. The spatial resolution of the Landsat data (excluding the thermal infrared band) was 30 m. In most situations, most of the surface feature’s width was less than 30 m. This means that a pixel in the Landsat images would possibily contain more than one type of small feature. The existence of mixed pixels was the main reason why the accuracy cannot measure up to traditional classification and area measurement [41]. The linear spectral un-mixing method which is based on sub-pixel un-mixing was possibily a better choice to solve the accuracy problem of the traditional classification. The linear spectral mixture analysis (LSMA) approach is based on the assumption that the spectrum was a linear combination of the spectral of all components in a pixel; the spectral proportions of the components represented the percentage of the surface features. It was also assumed that there was no interaction between the photons reflected by each component.

The linear spectral mixture analysis (LSMA) was adopted in this study for un-mixing pixels. The mathematical model of LSMA could be expressed as:

(2) $$R_{i}=\sum_{k=1}^{n}f_{k}\;R_{\mathrm{ik}}+{\mathrm{ER}}_{i}$$

where *i* = 1,…, m (number of spectral bands); *k* = 1,…, n (number of end members); R_i_ was the spectral reflectance of band i which contains one of more end members; f_k_ was the proportion of end member k within the pixel; R_ik_ was the known spectral reflectance of end member k within the pixel on band i; and ER_i_ was the error for band i. A constrained least-squares solution was used in this research, assuming that the following two conditions were satisfied simultaneously:

(3) $$\sum_{k=1}^{n}f_{k}=1$$

(4) $$0\leq f_{k}\leq 1$$

### 3.3. End Member Selection

End member selection was a key step in the LSMA approach. Many approaches have been developed for selecting end members such as the measurement spectrum based image based method. In this research, image based end member selection method was used because end members from images were easily obtained and they represented the spectra measured at the same scale as the image data [8].

One of the image based end-members selection methods was selecting representative homogeneous pixels from satellite images through visualizing spectral scatter plots of image band combinations [22]. Maximum Noise Fraction (MNF) was a helpful tool to guide image end member selection by putting almost 90% of the information on the first two or three components and thus minimizes the influence of band-to-band correlation [42], besides it could reduce the noise of the images. MNF transformation has two steps, firstly, it and reassigned the noise in signals; secondly it executed a principal component transition on white-noise data.

Having finished comparing the first three MNF components of each year, it was suggested that the spectra of the farm land was different from that of the forest and soil. Therefore, the five end members’ linear mixing model might be a better choice. In this research, five end members were selected: high albedo (e.g., new concrete, iron roofs of the factory), low albedo (e.g., old concrete roofs, asphalt roads), soil, forest, farm land (e.g., farm land, grass).

### 3.4. Impervious Surface Estimation

A constrained least-squares solution was applied to un-mix the MNF result into five fraction images. The vegetation was the sum of forest fraction and farm land fraction. Equation (5) was used to calculate the impervious surface coverage using high albedo and low albedo fraction images [7]. In this study, the effects of buildings shade and vegetation shade were ignored because they were insignificant to the ISA at this medium spatial resolution:

(5) $$R_{\mathrm{imp},b}=f_{\mathrm{low}}\;R_{\mathrm{low},b}+f_{\mathrm{high}}\;R_{\mathrm{high},b}+e_{b}$$

where *R_imp,b_* was the reflectance spectra of impervious surface of band *b*, *f_low_* and *f_high_* were the fraction of low albedo and high albedo, *R_low,b_* and *R_high,b_* were reflectance spectra of low and high albedo for band b. Equation (5) must meet the needs of the follow equations:

(6) $$f_{\mathrm{low}}+f_{\mathrm{high}}=1\;\text{and}\;f_{\mathrm{low}},\;f_{\mathrm{high}}>0$$

### 3.5. Accuracy Assessment

**Figure 2.**
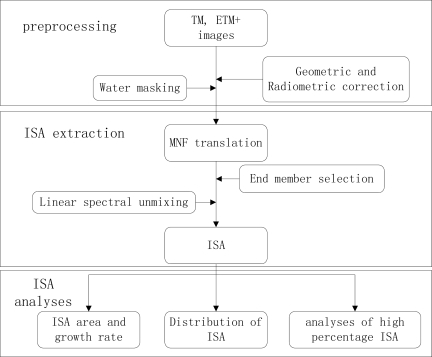
The sketch map of whole process flow of this study.

The accuracy assessment of impervious surface images was the process of checking out the quality of the results. Different elements of accuracy assessment, such as overall accuracy (OA), producer’s and user’s accuracy (PA and UA) and kappa coefficient, could be used and they could be calculated from an error matrix. Some researchers have discussed the approaches for accuracy assessment [43,44]. Root Mean Square Error (RMSE) and Systematic Error (SE) were utilized in this research to evaluate the accuracy of ISA estimation. RMSE and SE could be expressed as follows:

(7) $$\mathrm{RMSE}=\sqrt{\frac{\sum_{i=1}^{N}\left({\hat{X}}_{i}-X_{i}\right)}{N}}$$

(8) $$\mathrm{SE}=\frac{\sum_{i=1}^{N}\left({\hat{X}}_{i}-X_{i}\right)}{N}$$

where *X̂_i_* was sample i’s percentage of impervious surface estimated from TM and ETM+ data, and *X_i_* was sample i’s corresponding value of ground reference images, ***N*** is the number of the sample. The process flow of this study is shown in Figure 2.

## 4. Results and Discussion

### 4.1. ISA Area and Rate

The LSMA, one of the most popular methods in research of mixed-pixel un-mixing, was applied to estimate the percentage of ISA of 1998, 2003 and 2008. The LSMA results were constituted with several end-member images and a RSM error image. High resolution images of 1998, 2003 and 2008 from Google Earth’s history images were utilized as ground reference images for assessing the LMSA’s results. Random samples (650) were generated with the help of the ENVI “Generate Random Sample” tool. A 3 × 3 sampling unit was used to reduce the impacts of geometric errors associated with TM and Google Earth’s images. The percentage of the ISA in the high resolution image samples was calculated by visual interpretation. The results of accuracy assessment were shown in Table 1.

**Figure 3.**
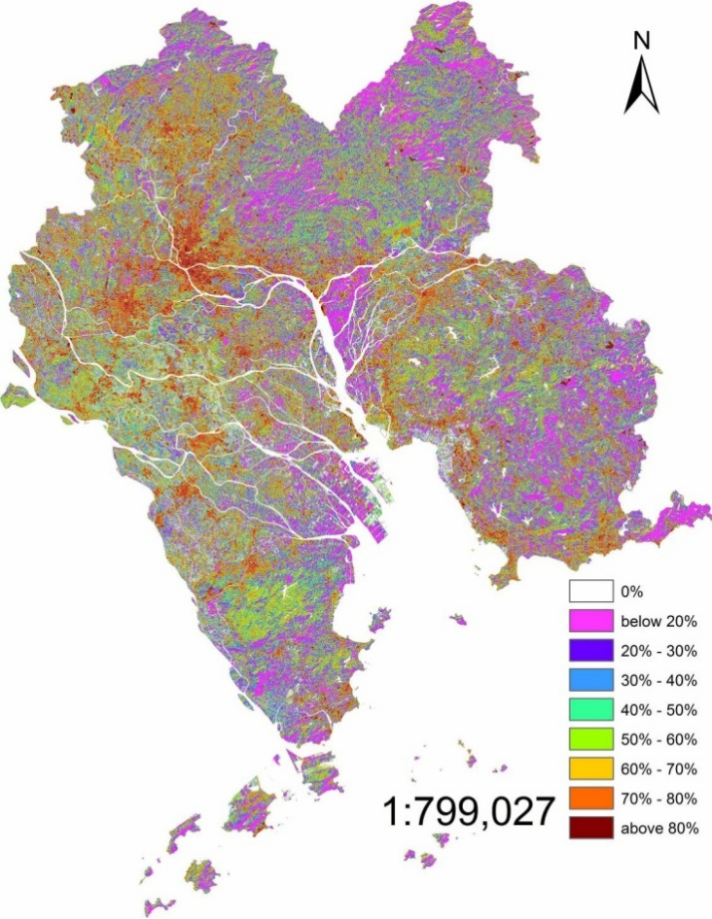
ISA percentage distribution of 1998.

**Figure 4.**
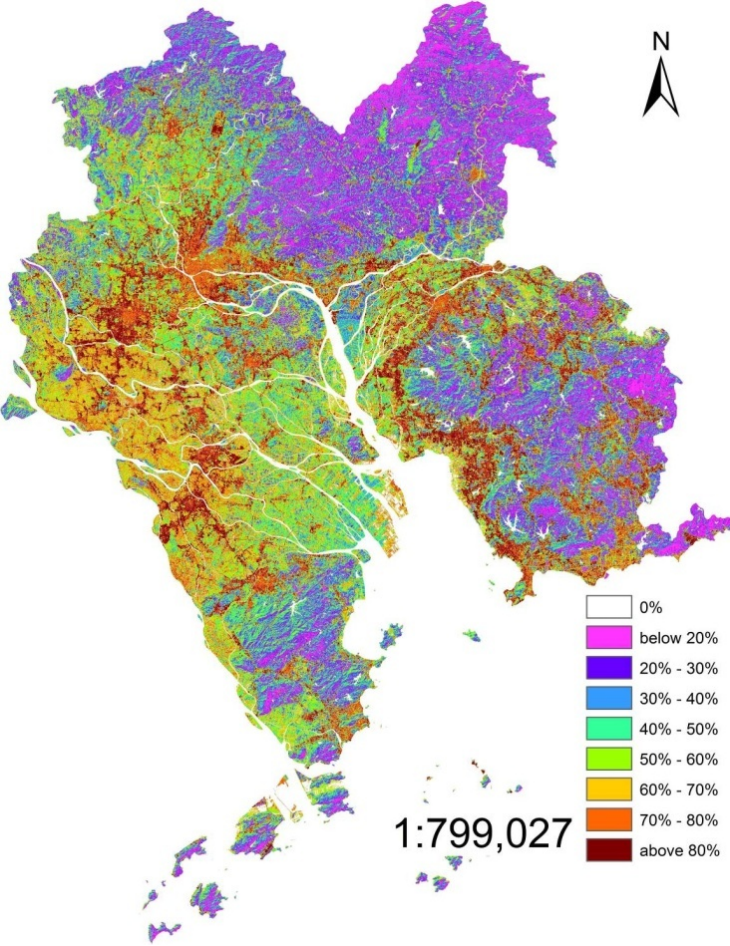
ISA percentage distribution of 2003.

**Figure 5.**
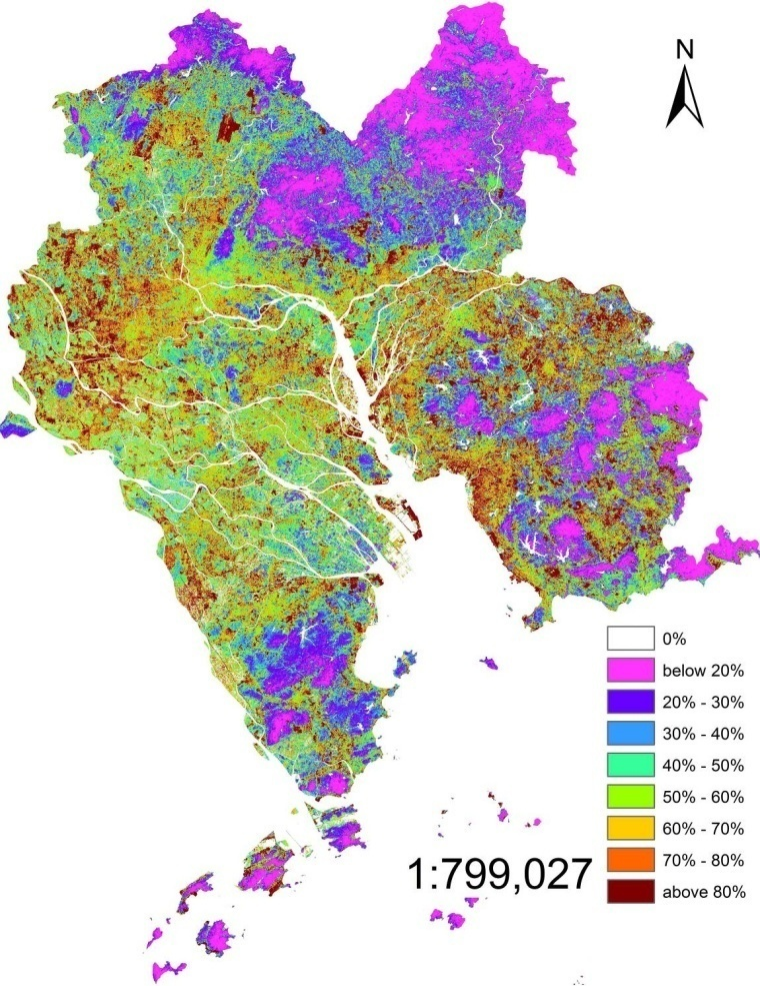
ISA percentage distribution of 2008.

The RSME were 0.20, 0.41 and 0.15 of 1998, 2003 and 2008, respectively. The ISA was calculated using Equations (5) and (6). The percentages of ISA in the core PRD (1998, 2003 and 2008) are shown in Figures 3–5. In these three figures, the same scale rule was applied and nine grades of ISA were differentiated according to percentage, and then, different colors were given to each grade.

In order to make clear and understand the ISA’s change, the temporal analyses, which could observe the long-term changed of the phenomena, were done following the factors of the area and percentage. The increased area/growth rate of ISA was calculated and discussed. The computed results are shown in Tables 1–2. From Tables 1–2, the past five years (1998–2008) had been suffering a rapid growth of the ISA from 5,080.76 km^2^ to 11,544.68 km^2^, while the increase in the first half of the decade was much more dramatic, 4,018 km^2^ in a mere five year period. Obviously, the growth rate in the period of 1998–2003 was relatively high at 79.09%, but it experienced a low increasing trend during 2003–2008 and the growth rate was 26.88%, only one third that of the 1998–2003 period.

### 4.2. Percentage Distribution of ISA

The surface imperviousness estimate was further classified into nine ranks based on the percentage of the ISA: (1) 0%; (2) <20%; (3) 20–30%; (4) 30–40%; (5) 40–50%; (6) 50–60%; (7) 60–70%; (8) 70–80%; (9) >80%. After the investigation and the comparison of the high resolution remote sensing images of the study area, it was found out that low percentage ISA, which was lower than 40%, mainly was located in the farmland and forest, such as northeastern, southeastern and southwestern core PRD. However, most of the middle percentage (40%–70%) and the high percentage (>70%) was located in the inner city and the fringes of the urban area. In addition, the middle percentage and the high percentage were the most closely connected with human activities. Therefore, the middle and high percentage, especially the high percentage, is discussed in detail. The high percentage ISA (>70%) in 1998 was mainly located in the north-western core PRD, especially in Guangzhou and Foshan districts which formed the biggest ISA core in the whole PRD. High percentage ISA in the southwestern core PRD presented a discrete pattern it mainly dominated the urban area. The distribution of the eastern core PRD were somewhat similar, high percentage ISA located in the urban areas of each city. Comparing to the ISA of Guangzhou and Foshan, the percentages of the eastern and southwestern core PRD were low and the area was small. It was different from the southwestern core PRD that had high percentage ISA in the east of Pearl River Estuary and north shore of Pearl River distributed as a ribbon by the influence of the road network. Middle percentage (40%–70%) ISA was mainly distributed in rural areas which were between the forest and the urban areas such as dry farm land, village and bare land. Western and northwestern core PRD was dominated by the middle percentage ISA.

The high percentage moved from inner centre to the rural edge in 2003. However, the percentage of inner city was lower than 1998, as was obvious in the Guangzhou and Foshan urban areas. The increased ISA expanded in a form of a branching that followed the extension of the road network in the rural areas at the edges of urban areas. The most typical area was the southwestern and southeastern core PRD. Besides, it was more clear that the high percentage ISA in eastern Pearl River Estuary was due to ribbon development.

The high percentage further lowered in the inner cities in 2008. Meanwhile, the highest percentage was located around the urban areas. Most of the rural area’s ISA percentage was higher than 2003 except for the forest areas. Southwestern core PRD was a typical case. The percentage of forest district changed a little, the percentage was still low there.

It was clearly seen from the ISA images of 2003 and 2008 that the middle and high percentage ISA was connected with the nearby cities in some region, such as Guangzhou and Foshan, Zhongshan and Shunde, Shenzhen and Dongguan. This was the result of the expansion of the high and middle percentage ISA.

Fan [31] has done some research about the urban areas of the core PRD and has classified the urban areas of 1998 and 2003 from the TM images. After a comparison of the results of Fan, it could be found that urban area was similar to the ISA which percentage was above 70%. The middle percentage ISA was mainly located in the paddy fields and some forest. The low percentage ISA was different from the high and middle percentage ISA, mainly located in the forests.

### 4.3. The Spatial-Temporal Change of the High Percentage ISA

**Figure 6.**
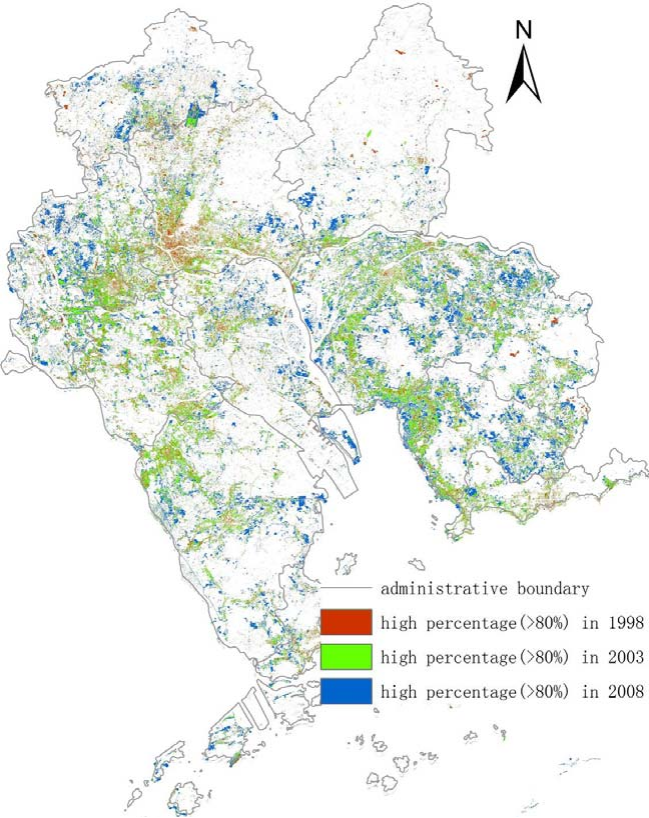
Spatial-temporal change of the high percentage ISA.

Figure 6 was made up of three data ISA images which were high percentage area of ISA in 1998, 2003, 2008. The bottom layer was the high percentage ISA image of 2008 which was colored in blue, middle layer was the image of 2003 which was shown in green color and the red layer revealed the information of high percentage ISA of 1998. Figure 6 showed that most of the high percentage ISA was located in the northwestern and southeastern core PRD in 1998, 2003 and 2008. The percentage of the northeastern and southwestern parts was relatively lower. The other characteristic of Figure 6 was that the high percentage ISA was moving from the inner cities to suburban areas. It was obvious in the region of eastern Dongguan and Shenzhen, northern Zhongshan and southern Shunde, especially in the Guangzhou and Foshan areas. High percentage ISA was mainly located in the inner urban areas (Guangzhou and Foshan inner city) in 1998. However, the distribution of the high percentage ISA had changed in that most of them had moved to the edges of the inner city which was the boundary between the urban and the suburban areas in 2003. On the one hand, it was the same as 2003, high percentage ISA in 2008 moved out of urban areas. On the other hand, it was different from 2003 in that the movement of high percentage ISA in 2008 blended with healthy and gradual change. The distribution of high percentage ISA of 2003 and 2008 illustrated that the movement of the western core PRD moved mainly in the form of healthy change while the eastern core had a gradual change. Northwestern and Southwestern core PRD could be a very good example of the healthy change that high percentage ISA of 2008 was at a distance of 2003’s. Meanwhile, the southeastern core PRD might be a good instance of the gradual change in that the high percentage ISA of this core in 2008 was connected with the ISA of 2003. Eastern Dongguan was a typical area of the gradual change. For the whole region, the high percentage ISA moved outward from the centre line of the Pearl River. The ISA of the western PRD was westward while the ISA of the eastern PRD was eastward. It could be noted that the high percentage ISA of the eastern and western PRD changed from a cluster into a large cluster from 1998 to 2008. However, high percentage ISA on both shores of the Pearl River Estuary moved toward the Pearl River. Especially in northwestern Shenzhen and southwestern Dongguan, the ISA there expended toward the Pearl River shoreline.

## 5. Discussion

The LSMA which was used for extracting the information of ISA was a useful method in the mix-pixel researches. For the middle solution remote sensing image (TM 5 or Hyperion), the LSMA method improved the accuracy of classification because it assumed that a pixel was composed of several surface features and the LSMA’s result was the percentage of each surface feature. Compared to the pixel based classification method, the LSMA’s result was closer to the actual ground. The results of 1998, 2003 and 2008 depicted the ISA’s distribution of core PRD and the high percentage ISA’s location was shown in Figures 3–6. ISA of core PRD has experienced a fast expansion period from 1998 to 2008. The middle and high percentage extended from the inner cities to suburban areas and the inner cities’ ISA percentage became lower and lower during this period. However, the percentage of ISA was becoming higher and higher for the whole region.

The high percentage ISA of 1998 was different from 2003 and 2008 that it mainly located in the inner cities. This might related to the Asian financial crisis which appeared at 1997. The crisis had great negative impact on economic development that slowed down the speed of urbanization. The spatial change of cities focused on the renewal of the inner area instead of the edges of the urban area during this period.

After 2000, the land prices in the inner cities were becoming more and more expensive. Some industries were transferred to the districts where the land prices were lower. Besides, the government of Guangdong promoted the transfer of industries. As a result, a large number of factories moved to suburban areas, which was shown at the ISA images of 2003 and 2008 where the high percentage ISA was mainly located in suburban areas. Moreover, under the influence of the APEC, the regional economy of China had a fast development. The organization of “9 + 2” (Guangdong, Fujian, Jiangxi, Hunan, Guangxi, Hainan, Sichun, Yunnan, Guizhou and HongKong, Macao) had formed which propelled the transfer of the industries directly. Furthermore, after joining the WTO, the Chinese economy had experienced a rapid growth period, especially in the Pearl River Delta, and urban areas expanded rapidly.

Major sporting events had some impact on the distribution of high percentage ISA. The 16th Asian Games were held in Guangzhou at 2010 and some sport events were held in Foshan and Dongguan. In addition, the 26th Universiade was held in 2011 in Shenzhen. Large infrastructure was improved before the games began. What is more, most of the venues were located outside the inner city that might contribute some part of high percentage ISA in suburban areas.

Finally, the topography also had certain impact on the distribution of high percentage ISA. The northeastern and southwestern core PRD was a typical example. Most of northeastern and southwestern core PRD was mountains which are not suitable for building. Hence, little high percentage ISA was located there.

However, other issues about extracting and analyzing ISA using remote sensing data still were not discussed in this study. (1) ISA was estimated higher in suburban areas. Fishponds are similar to the low albedo and the dry soil was similar to the high albedo. Therefore, the ISA images of the suburban parts, especially the fishponds and the dry soil may overestimated; (2) ISA was estimated lower in inner cities. Dark impervious surfaces such as shadows of tall building in the inner city were similar with the water. It was inevitable that some dark impervious surfaces would be missed while masking the water areas.

## 6. Conclusions

The most popular sub-pixel un-mixing method, the linear spectral un-mixing method was chosen to extract the information of ISA of the core PRD based on time-series remote sensing data. The ISA areas of three years (1998/2003/2008) were obtained and analyzed. (1) The ISA increased 4,018.24 km^2^ from 5,080.76 in 1998 to 9,099.00 in 2003 with a growth rate of 79.09%. During 2003–2008 the ISA growth rate came down, the area increased 2,445.69 km^2^ and the growth rate was 26.88%; (2) The temporal sequence of percentage ISA maps showed an increased middle and high percentage ISA and a decreased low percentage ISA throughout the study period in the whole core PRD. Increased connectivity of middle and high percentage ISA of nearby cities was the result of expansion of ISA in suburban areas. The middle and high percentage ISA was mainly located in the northwestern and southeastern core PRD, especially in the Guangzhou and Foshan districts. After the comparison of the existing research results, it found out that urban areas were similar with the ISA whose percentage was higher than 70%; (3) The high percentage ISA in the southeastern, northwestern, western and northern regions had experienced a period of rapid development from 1998 to 2003. High percentage ISA moved from the inner city to edges of the urban areas during 1998–2003 and moved to the suburban areas far from the urban area jumpily during 2003–2008. According to the discussion of high percentage ISA spatial expanded direction, it could be found out that high percentage ISA moved outward from the centre line of the Pearl River while high percentage ISA in both shores of the Pearl River Estuary moved toward the Pearl River; (4) ISA percentage change could be related with the regional economic development. Also, major sport events and the government policies could promote the development of the ISA. Meanwhile, topography did limit to some extent the expansion of the ISA.

## Figures and Tables

**Table 1.** Percentage of ISA and the accuracy of 1998, 2003 and 2008.

**ISA**	**Area (km^2^)**	**Percentage**
1998	5080.76	13.16%
2003	9099.00	23.57%
2008	11544.68	29.48%

**Table 2.** The increasing area and growth rate of 2003–1998 and 2008–2003.

**ISA**	**Increasing Area (km^2^)**	**Growth Rate of 5 Years**
1998–2003	4,018.24	79.09%
2003–2008	2,445.69	26.88%
